# Supplementary material for: Diminishing effects of mechanical loading over time during rat Achilles tendon healing
Source: PLoS One. 2020 Dec 14;15(12):e0236681. doi: 10.1371/journal.pone.0236681 (PMC7735574; doi:10.1371/journal.pone.0236681)
Supplement: S1 Fig — (PDF) [file pone.0236681.s001.pdf]

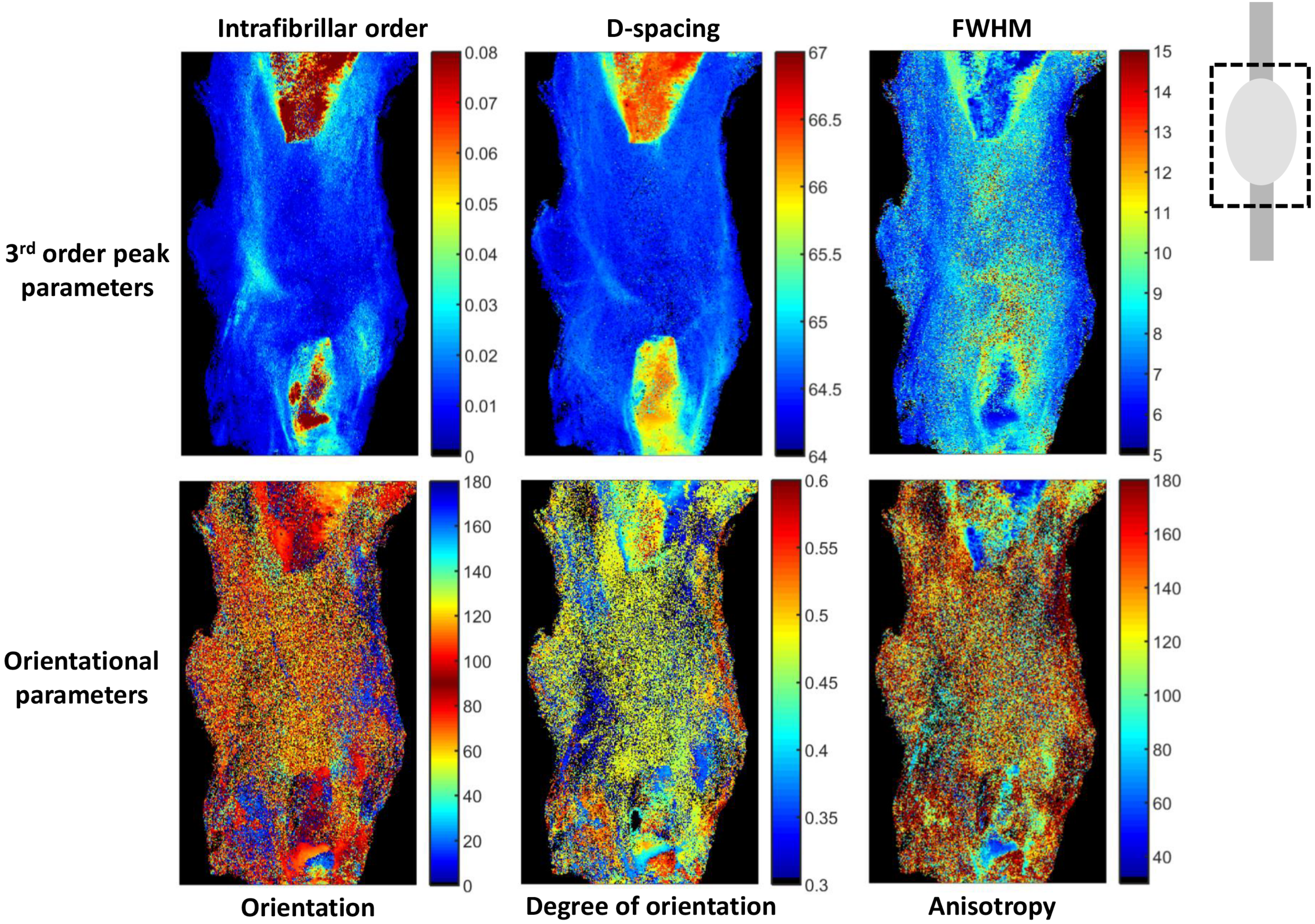

SUPPLEMENTARY Figure 1: Example mapping of a healing tendon after 1 week, showing the maps of the different parameters that were quantified. Intrafibrillar order (AU), D-spacing (nm) and FWHM (nm) are determined from the 3rd order peak. Orientation (degree), degree of orientation (AU) and anisotropy (degrees) are the orientational parameters.
