# Supplementary material for: Diminishing effects of mechanical loading over time during rat Achilles tendon healing
Source: PLoS One. 2020 Dec 14;15(12):e0236681. doi: 10.1371/journal.pone.0236681 (PMC7735574; doi:10.1371/journal.pone.0236681)
Supplement: S2 Fig — (PDF) [file pone.0236681.s002.pdf]

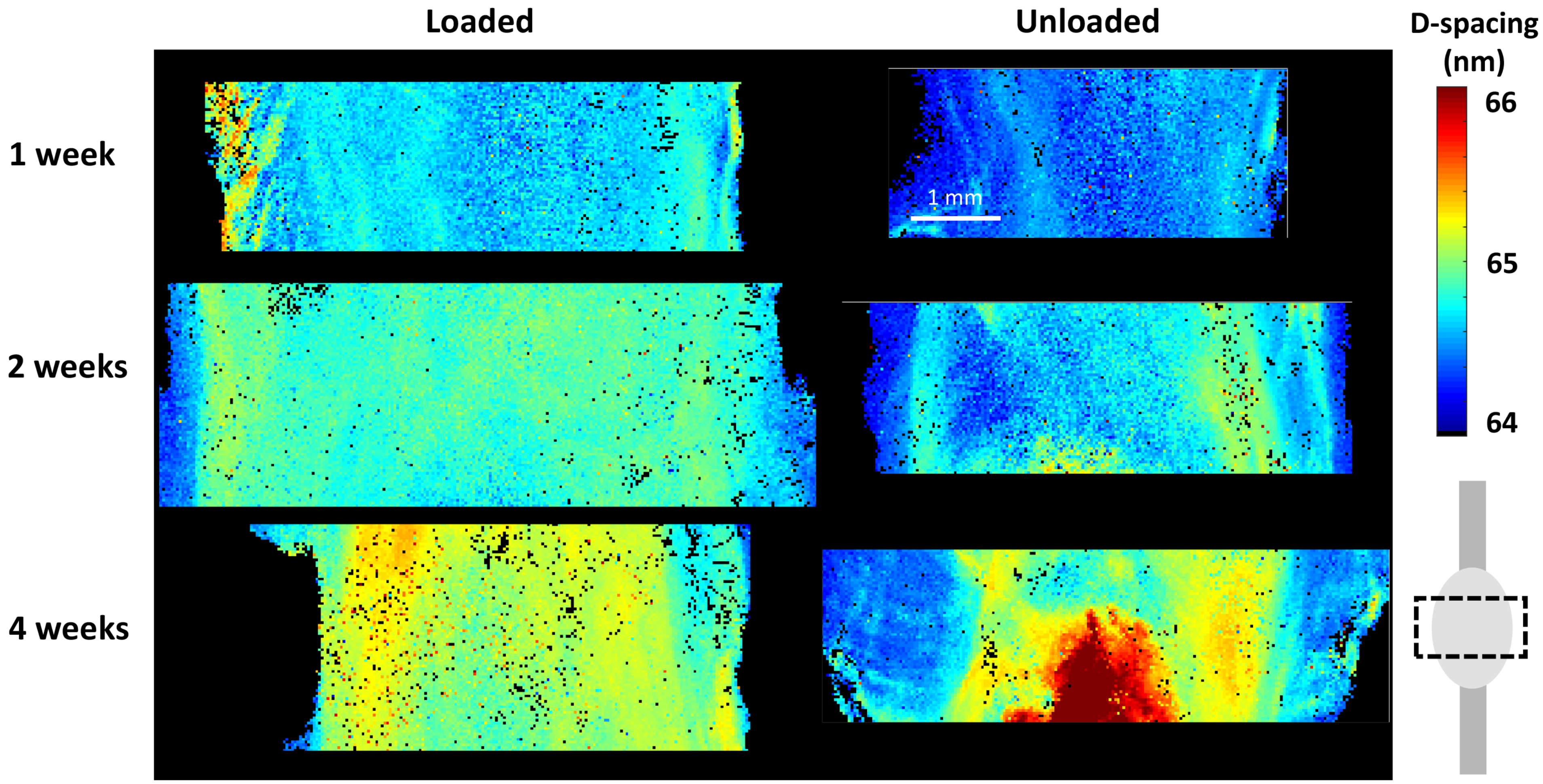

SUPPLEMENTARY Figure 2: SAXS mapping of the D-spacing parameter in the callus region in representative loaded and unloaded samples at 1, 2 and 4 weeks of healing.
