# Supplementary material for: Diminishing effects of mechanical loading over time during rat Achilles tendon healing
Source: PLoS One. 2020 Dec 14;15(12):e0236681. doi: 10.1371/journal.pone.0236681 (PMC7735574; doi:10.1371/journal.pone.0236681)
Supplement: S1 Table — Data is represented as mean (standard deviation). (PDF) [file pone.0236681.s003.pdf]

**SUPPLEMENTARY Table 1:** Summary of all mechanical testing data. Data is represented as mean (standard deviation).

| | | W1 | | W2 | | W4 | |
|---|---|---|---|---|---|---|---|
| | | **Normal loading** | **Unloading** | **Normal loading** | **Unloading** | **Normal loading** | **Unloading** |
| | N | 10 | 9 | 10 | 10 | 10 | 10 |
| Callus length (mm) | | 5.5 (1.5) | 4.1 (0.6) | 7.1 (2.7) * | 4.6 (2.3) * | 4.9 (1.5) $^{\Psi}$ | 3.8 (1.2) |
| Cross-sectional area ($mm^2$) | | 23.3 (6.4) ** | 10.7 (2.9) ** | 20.8 (6.5) | 16.6 (6.1) $^{\Psi}$ | 17.8 (3.9) | 15.6 (3.9) |
| Creep magnitude (mm) | 5N creep | 2.0 (0.3) * | 1.1 (0.1) * | 1.7 (0.5) * | 1.4 (0.4) * | 1.5 (0.3) # | 1.4 (0.4) |
| | 12N creep | 1.7 (0.4) | - | 1.4 (0.4) | 0.6 (0.4) | 1.2 (0.2) # | 1.1 (0.3) $^{\Psi}$ |
| Creep ratio (%) | 5N creep | 40 (16) * | 28 (3) * | 25 (6) * $^{\Psi}$ | 33 (9) * | 33 (8) | 37(4) # |
| | 12N | 41 (14) | - | 29 (20) | 32 (7) | 27 (6) | 29 (5) |
| Stiffness (N/mm) | 5N creep | 3.1 (0.4) ** | 4.9 (0.7) ** | 4.2 (1.4) | 4.9 (1.7) | 4.5 (2.0) | 4.4 (1.0) |
| | 12N creep | 6.8 (1.0) | - | 10.4 (2.4) | 10.2 (2.6) | 6.1 (1.5) | 11.0 (2.8) |
| | Load to failure | 14.5 (4.5) * | 7.8 (1.0) * | 22.5 (3.9) $^{\Psi}$ | 21.7 (4.1) | 28.0 (4.4) ### | 25.8 (6.1) ### |
| Young's modulus (MPa) | 5N creep | 0.8 (0.4) * | 2.0 (0.7) * | 1.4 (0.5) | 1.3 (0.4) | 1.2 (0.4) | 1.1 (0.4) ## |
| | 12N creep | 2.2 (1.1) | - | 3.1 (0.9) | 2.8 (0.9) | 2.4 (0.4) | 2.7 (0.8) |
| | Load to failure | 3.9 (2.0) | 3.2 (1.0) | 6.9 (1.8) | 6.1 (1.7) $^{\Psi\Psi}$ | 7.6 (2.0) ## | 6.5 (2.2) ### |
| Peak force (N) | | 24.1 (6.4) ** | 11.4 (1.0) ** | 46.8 (20.0) * | 27.0 (4.9) * $^{\Psi}$ | 60.2 (17.9) ## | 55.0 (18.2) $^{\Psi\Psi}$ ### |
| Peak stress (MPa) | | 1.1 (0.3) | 1.1 (0.3) | 2.2 (0.6) $^{\Psi\Psi}$ | 1.9 (0.6) $^{\Psi}$ | 3.4 (0.6) $^{\Psi\Psi}$ ### | 3.6 (0.9) $^{\Psi\Psi\Psi}$ ### |

* $p < 0.05$, ** $p < 0.01$, *** $p < 0.001$
* significant difference between normal loading and unloading by botox, within the same time point
# significant difference between week 1 and 4, within the same treatment group
$^{\Psi}$ significant difference between this and previous time point, within the same treatment group
