# Supplementary material for: Diminishing effects of mechanical loading over time during rat Achilles tendon healing
Source: PLoS One. 2020 Dec 14;15(12):e0236681. doi: 10.1371/journal.pone.0236681 (PMC7735574; doi:10.1371/journal.pone.0236681)
Supplement: S2 Table — Data is represented as mean (standard deviation). (PDF) [file pone.0236681.s004.pdf]

SUPPLEMENTARY Table 2: Summary of results from SAXS analysis from the full tendon and the separate regions of interest. Data is represented as mean (standard deviation).

		Week 1		Week 2		Week 4	
		**Normal loading**	**Unloading**	**Normal loading**	**Unloading**	**Normal loading**	**Unloading**
	N	7	8	6	7	6	7
D-spacing (nm)	Full tendon	64.63 (0.03)	64.54 (0.02)	64.88 (0.02)	64.80 (0.06)	65.04 (0.04)	65.09 (0.19)
	Lateral	64.57 (0.06)	64.54 (0.05)	64.89 (0.02)	64.73 (0.07)	65.05 (0.07)	65.07 (0.19)
	Centre	64.70 (0.02)	64.60 (0.06)	64.86 (0.04)	64.86 (0.06)	65.07 (0.03)	65.06 (0.10)
	Medial	64.70 (0.02)	64.60 (0.06)	64.86 (0.06)	64.86 (0.04)	65.07 (0.03)	65.06 (0.10)
Intrafibrillar order (AU)	Full tendon	0.017 (0.001)	0.016 (0.002)	0.022 (0.005)	0.018 (0.005)	0.048 (0.016)	0.038 (0.020)
	Lateral	0.016 (0.002)	0.016 (0.004)	0.020 (0.004)	0.015 (0.005)	0.041 (0.014)	0.033 (0.017)
	Centre	0.014 (0.002)	0.012 (0.001)	0.022 (0.004)	0.013 (0.001)	0.039 (0.011)	0.037 (0.024)
	Medial	0.020 (0.003)	0.020 (0.005)	0.026 (0.008)	0.023 (0.002)	0.063 (0.027)	0.044 (0.021
Degree of orientation (AU)	Full tendon	0.42 (0.01)	0.44 (0.01)	0.45 (0.02)	0.45 (0.02)	0.46 (0.01)	0.46 (0.02)
	Lateral	0.42 (0.02)	0.43 (0.02)	0.45 (0.01)	0.45 (0.02)	0.46 (0.01)	0.46 (0.02)
	Centre	0.42 (0.01)	0.43 (0.02)	0.44 (0.02)	0.45 (0.02)	0.46 (0.04)	0.46 (0.04)
	Medial	0.43 (0.01)	0.45 (0.02)	0.47 (0.03)	0.46 (0.04)	0.45 (0.01)	0.47 (0.02)
FWHM (nm)	Full tendon	7.60 (0.23)	7.60 (0.23)	8.17 (0.40)	8.86 (0.47)	8.32 (0.25)	8.05 (0.45)
	Lateral	7.77 (0.31)	7.39 (0.37)	8.17 (0.48)	8.85 (0.36)	8.35 (0.28)	8.22 (0.49)
	Centre	8.03 (0.42)	8.40 (0.35)	8.62 (0.41)	9.79 (0.77)	8.60 (0.27)	8.45 (0.44)
	Medial	6.98 (0.24)	7.38 (0.60)	7.69 (0.55)	7.88 (0.48)	8.02 (0.24)	7.46 (0.50)
Anisotropy (degrees)	Full tendon	138.2 (4.5)	135.6 (4.9)	142. 8 (7.0)	134.0 (3.2)	125.0 (16.4)	127.7 (10.6)
	Lateral	134.7 (8.8)	133.3 (8.0)	143.8 (2.7)	143.2 (5.2)	128.3 (15.0)	131.1 (9. 8)
	Centre	136.6 (6.4)	136.3 (5.2)	144.6 (6.9)	140.4 (8.0)	137.6 (17.9)	133.9 (17.8)
	Medial	142.6 (7.8)	137.0 (6.5)	139.7 (15.2)	120.1 (10.5)	109.4 (22.4)	117.7 (17.8)
